# Preliminary study on BRICS sequential therapeutic regimen as salvage treatment for refractory advanced colorectal cancer patients harboring pMMR status

**DOI:** 10.3389/fimmu.2025.1670812

**Published:** 2025-09-18

**Authors:** Yonghai Peng, Jian Wang, Junhui Wang, Jianxin Chen

**Affiliations:** ^1^ Department of Oncology, Cangshan Hospital Area, 900 Hospital of the Joint Logistics Support Force, Fujian, Fuzhou, China; ^2^ Department of Gastroenterology, Jiaxing Second Hospital, Jiaxing, Zhejiang, China; ^3^ Department of Radiation, The Quzhou Affiliated Hospital of Wenzhou Medical University, Quzhou People′s Hospital, Quzhou, Zhejiang, China; ^4^ Department of Education, International Word, The Quzhou Affiliated Hospital of Wenzhou Medical University, Quzhou People′s Hospital, Quzhou, Zhejiang, China

**Keywords:** metastatic colorectal cancer, mismatch repair-proficient, BRICS regimen, salvage therapy, stereotactic body radiotherapy

## Abstract

**Background:**

Patients with refractory mismatch repair-proficient/microsatellite stable (pMMR/MSS) metastatic colorectal cancer exhibit intrinsic resistance to immune checkpoint inhibitors and conventional salvage therapies, with median overall survival (OS) is typically less than 10 months. This study evaluates the novel BRICS sequential regimen (Bifidobacterium supplementation, Radiotherapy, Immunotherapy, Chemotherapy, Stereotactic approach) in this population.

**Methods:**

In this retrospective analysis, 27 refractory pMMR/MSS metastatic CRC patients received BRICS: Stereotactic body radiotherapy to a single lesion, indefinite high-dose probiotics, followed by low-dose chemotherapy plus PD-1 inhibitor. Primary endpoints were objective response rate (ORR), disease control rate (DCR), progression-free survival (PFS), and OS.

**Results:**

Efficacy outcomes: ORR 33.3% (9 partial responses), DCR 88.9%, median PFS 7.20 months (95% CI: 5.23–9.18), and median OS 12.30 months (95% CI: 9.89–14.71). Eastern Cooperative Oncology Group Performance Status (ECOG PS) >2 independently predicted inferior OS (HR = 4.860; p=0.042), while metastatic burden (≥3 organs) predicted shorter PFS (HR = 3.179; p=0.026). Grade 3–4 adverse events occurred in 22.2% of patients (neutropenia: 14.8%; thrombocytopenia: 3.7%; anemia: 3.7%).

**Conclusions:**

BRICS demonstrates clinically meaningful efficacy (DCR 88.9%, mOS 12.3 months) and manageable toxicity in refractory pMMR/MSS metastatic CRC. ECOG PS >2 and high metastatic burden identify patients with limited benefit, warranting prospective validation in biomarker-enriched cohorts.

## 1 Introduction

Colorectal cancer (CRC) remains the third most common malignancy and second leading cause of cancer-related mortality globally, with over 1.9 million new cases and 930,000 deaths annually (1). For patients with advanced mismatch repair-proficient (pMMR) or microsatellite stable (MSS) metastatic CRC-representing approximately 85% of cases-the therapeutic landscape remains particularly challenging (2). Despite standard chemotherapeutic regimens and targeted therapies, long-term outcomes are poor, with median overall survival (OS) below 10 months in refractory cases (3). Crucially, refractory pMMR/MSS metastatic CRC exhibits intrinsic resistance to immune checkpoint inhibitors (4). The KEYNOTE-016 trial underscored this limitation, demonstrating no significant progression-free survival (PFS) benefit with pembrolizumab monotherapy in pMMR/MSS metastatic CRC (5).

Recent advances in immunotherapy have explored multimodal strategies to overcome ICI resistance. Stereotactic body radiotherapy (SBRT) has emerged as a potent immunomodulator, inducing immunogenic cell death and releasing tumor neoantigens that promote dendritic cell activation and T-cell priming (6). Clinical evidence from the PEMBRO-RT trial revealed that combining SBRT with anti-PD-1 therapy doubled objective response rates in PD-L1-negative non-small cell lung cancer (NSCLC) compared to ICI monotherapy (ORR: 36% *vs*. 18%) (7). Concurrently, the gut microbiome-particularly Bifidobacterium species-has been shown to enhance ICI efficacy by modulating systemic immunity through CD8+ T-cell activation and dendritic cell maturation (8, 9). Translational studies further indicate that probiotic supplementation may augment anti-tumor responses in solid malignancies (10, 11). Additionally, low-dose metronomic chemotherapy exerts immunostimulatory effects by depleting immunosuppressive regulatory T cells (Tregs) and myeloid-derived suppressor cells (MDSCs) while preserving effector T-cell populations (12).

Building upon these principles, our team pioneered the ​BRICS sequential therapeutic regimen-an acronym for ​Bifidobacterium supplementation, ​Radiotherapy, ​Immunotherapy (PD-1 inhibitors), ​Chemotherapy (low-dose), and ​Stereotactic approach. This strategy temporally coordinates SBRT-induced antigen release, microbiome modulation, and metronomic chemotherapy to sustain immune activation. In a recent retrospective study of PD-L1-negative, EGFR/ALK wild-type metastatic NSCLC (n=23), BRICS achieved unprecedented efficacy: objective response rate (ORR) 95.7%, median PFS 16.0 months (95% CI: 9.11–22.89), and median OS 32.7 months (95% CI: 11.53–53.87) with minimal toxicity (13). These results demonstrated the regimen’s capacity to convert immunologically cold tumors into responsive microenvironments.

Given the mechanistic parallels between PD-L1-negative NSCLC and pMMR CRC-both characterized by T-cell exclusion and ICI resistance—we hypothesized that BRICS could address the critical unmet need in refractory pMMR/MSS metastatic CRC. This preliminary study aims to evaluate the efficacy and safety of the BRICS regimen in this population, leveraging our established protocol to potentially redefine therapeutic paradigms for immunotherapy-resistant gastrointestinal malignancies.

## 2 Methods

### 2.1 Data source

The data of patients diagnosed as pMMR advanced colorectal cancer patients at Cangshan Hospital Area, 900 Hospital of the Joint Logistics Support Force, and Quzhou People’s Hospital between January 2018 and December 2024 were retrieved from the electronic medical record system. Patients were deemed eligible for inclusion in this retrospective real-world study if they met the following criteria: (1) a definitive histological or cytological diagnosis of advanced colorectal cancer; (2) immunohistochemically confirmed pMMR status or NGS-confirmed MSS status.; (3) absence of treatable target mutations such as Her-2, B-raf, or KRAS wild type; (4) receipt of the ‘BRICS’ treatment strategy; (5) presence of at least one measurable lesion; and (6) patients who had disease progression or intolerability after receiving at least ​two prior lines​ of systemic therapy ​including oxaliplatin, irinotecan, and fluorouracil. In addition, the exclusion criteria were as follows: (1) a history of autoimmune disease; (2) a poor ECOG performance status of > 3. (3). without measurable lesions. The follow-up deadline was set for March 31^th^, 2025. This retrospective analysis consecutively enrolled all refractory pMMR/MSS metastatic CRC patients meeting the inclusion criteria at the above institutions, with no exclusion based on perceived benefit from BRICS. While this approach minimizes selection bias, the retrospective nature may still influence outcome interpretation, and we acknowledge this limitation in the discussion. This study received approval from the Ethics Committee of Quzhou People’s Hospital and the Ethics Committee of 900 Hospital of the Joint Logistics Support Force, and all investigations were conducted in accordance with the Declaration of Helsinki (revised in 2013).

### 2.2 Treatment procedure

**Figure 1:**
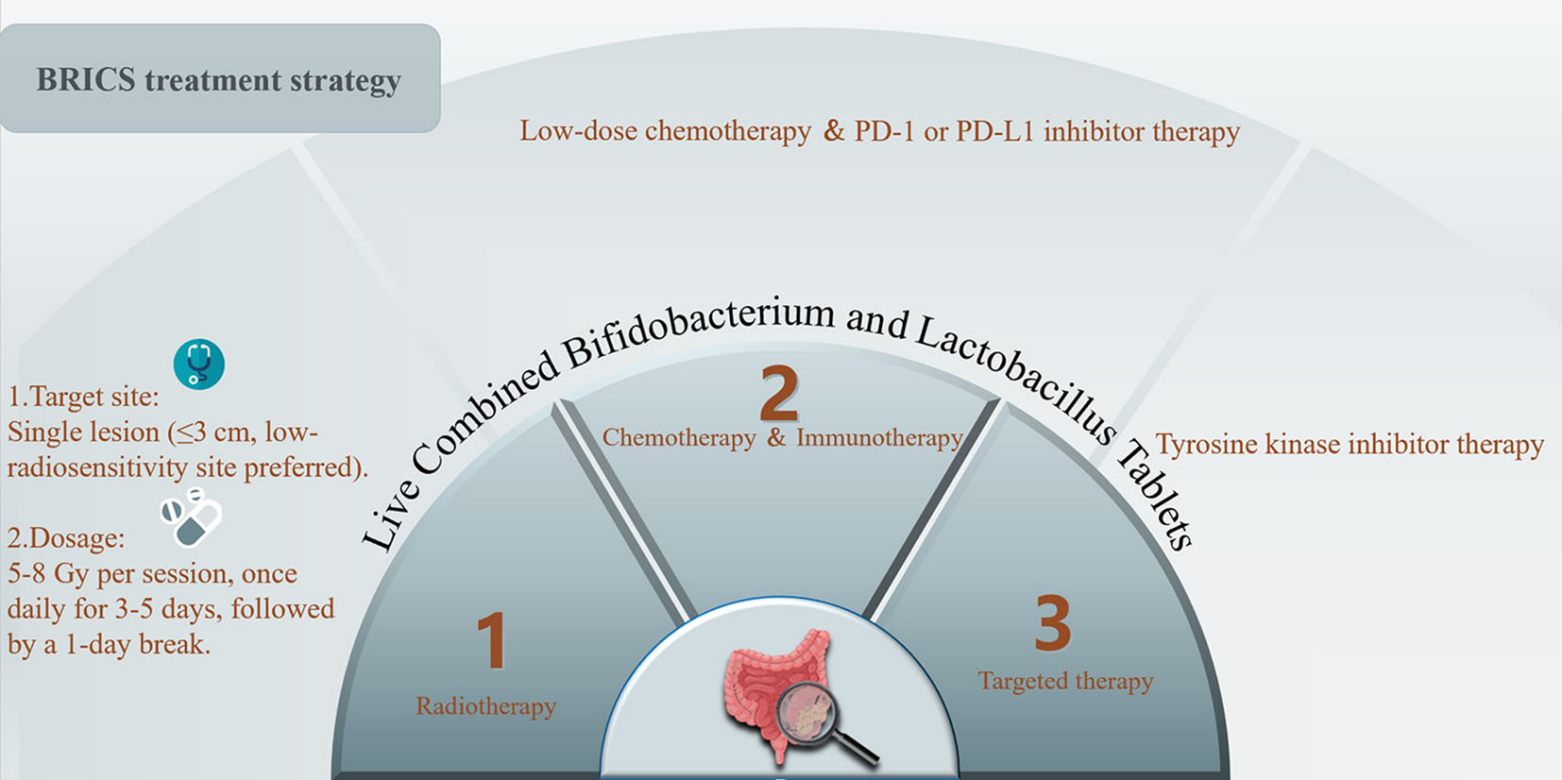
Treatment protocol of BRICS sequential therapeutic regimen.

The BRICS therapeutic regimen was follows: Eligible patients underwent stereotactic body radiotherapy (SBRT) targeting a single metastatic lesion (preferably ≤3 cm, anatomically well-circumscribed regions such as intrahepatic metastatic lesions) with 5–8 Gy delivered daily over three to five consecutive days (based on the size, and location of the tumor for radiation). SBRT technical specifications:​​ Stereotactic body radiotherapy (SBRT) was delivered using ​volumetric modulated arc therapy (VMAT)​. The prescription dose ranged from ​25–40 Gy in 3–5 fractions, adjusted based on lesion location. The biologically effective dose (BED) was calculated with an ​α/β ratio of 10 Gy​ for tumor control. Organ-at-risk constraints adhered to AAPM TG-101 guidelines: ​Spinal cord: Cumulative dose limit of ​​<22 Gy to 0.35 cm³​. ​Liver: ​**>700 cc receiving <21.5 Gy**​. ​Lungs: ​**>1500 cc (male) or >950 cc (female) receiving <12.5 Gy**. Concurrently, oral triple-dose Bifidobacterium/Lactobacillus probiotics (Bifidobacterium longum​​ (Strain No.: S19980004) at a dose of ​2×10^8 CFU/day, administered ​until disease progression or intolerable toxicity) were initiated and continued indefinitely. After a 24-hour post-SBRT recovery period, patients received low-dose chemotherapy (such as low-dose FOLFOX regimen: oxaliplatin 100mg d1 iv + 5-fluorouracil 500mg iv once, 1.5g civ 48 hours + calcium folinate 500mg d1 iv/every 21 days) combined with a PD-1 inhibitor, repeated every 21 days for up to six cycles. Chemotherapy dose reductions were implemented for grade ≥3 hematologic toxicity, while immune-related adverse events were managed per ASCO guidelines. Following completion of six cycles, patients continued probiotic maintenance while undergoing quarterly surveillance with contrast-enhanced CT/MRI monitoring. In cases of disease progression, a new cycle of BRICS therapy was initiated by selecting one progressing lesion meeting the original SBRT criteria (≤3 cm, non-critical location) for re-irradiation. Post-SBRT, the patient received an additional up to six cycles of BRICS chemoimmunotherapy, followed by treatment discontinuation and surveillance. This iterative approach continued until systemic progression or intolerance, with cumulative radiation doses constrained to organ-at-risk tolerance limits (e.g., spinal cord cumulative Dmax <45 Gy). The protocol prioritized temporal coordination of SBRT-induced antigen release, microbiome modulation, and metronomic chemotherapy to sustain immune activation. The treatment protocol was presented in the **Figure 1**.

### 2.3 Data collection and outcomes evaluations

Clinical response to the BRICS sequential therapeutic regimen was evaluated according to the Response Evaluation Criteria in Solid Tumors (RECIST) version 1.1. The enrolled patients underwent imaging evaluation every 6–8 weeks during treatment, while every 12 weeks after treatment. The objective response rate (ORR) was defined as the percentage of patients who achieved a complete response (CR: complete remission of all target lesions) or partial response (PR: at least a 30% reduction in the sum of the diameters of target lesions). Progressive disease (PD) referred to a 20% increase in the sum of the diameters of target lesions. A disease that could not be classified as PR or PD was evaluated as stable disease (SD). The percentage of patients with CR, PR, or SD was defined as the disease control rate (DCR). PFS was calculated as the time from the initiation treatment of BRICS to PD or death. OS referred to the time from the initiation treatment of BRICS to death of the deadline of the follow-up. Adverse events (AEs) were graded according to the National Cancer Institute Common Terminology Criteria for Adverse Events version 4.0 (NCI-CTCAE v4.0).

### 2.4 Statistical analysis

Descriptive statistics (percentages, means, and medians) were used to describe the baseline characteristics and clinical features of the patients with advanced colorectal cancer. Short-term efficacy was evaluated using ORR and DCR. Survival curves were calculated using the Kaplan-Meier method and were compared via the log-rank test based on ECOG PS and metastatic organs. Multivariable Cox regression was considered but not performed due to the limited sample size. K-M curves were plotted using GraphPad Prism 9.0 (GraphPad Software Inc., San Diego, CA, USA). These analyses were performed using SPSS software, version 23.0 (SPSS Inc., Chicago, USA). P ≤ 0.05 was considered to indicate statistical significance.

## 3 Results

### 3.1 Patient characteristics and outcomes

A total of 27 patients were enrolled in this study. Baseline characteristics are summarized in **Table 1**. The median age was 62 years (range: 45-75), with 66.7% (n=18) aged ≥60 years. Gender distribution was approximately balanced (44.4% male, 55.6% female). All patients (100.0%) presented with TNM stage IV disease. The majority were non-smokers (74.1%), and primary tumors originated predominantly in the colon (59.3%). Metastatic burden involved <3 organs in 63.0% of patients and ≥3 organs in 37.0%. Most patients (77.8%) had an ECOG performance status ≤2. Prior treatment lines included second-line therapy (29.6%) and third-line or beyond therapy (70.4%). Within the BRICS regimen, the most frequently irradiated site was the liver (51.9%). Immunotherapy agents comprised Toripalimab (25.9%), Sintilimab (25.9%), and Camrelizumab (18.5%). Most patients (63.0%) received <6 cycles of BRICS therapy.

**Table 1:** Baseline characteristics.

Baseline characteristics	All patients (n = 27)
Age (years), n (%)	
Median (range)	62 (45-75)
≥60	18 (66.7)
<60	9 (33.3)
Gender, n (%)	
Male	12 (55.6)
Female	15 (44.4)
TNM stage, n (%)	
III	0 (0)
IV	27 (100.0)
Smoking status, n (%)	
Nonsmoker	20 (74.1)
Former smoker/smoker	7 (25.9)
Primary tumor site, n (%)	
Colon	16 (59.3)
Rectum	11 (40.7)
Number of metastatic organs, n (%)	
< 3	17 (63.0)
≥ 3	10 (37.0)
ECOG PS, n (%)	
≤ 2	21 (77.7)
> 2	6 (22.3)
Number of prior lines of standard therapy, n (%)	
Second-line therapy	8 (29.6)
Third-line and beyond therapy	19 (70.4)
Prior systemic therapies, n (%)	
Fluoropyrimidine-based	27 (100%)
Oxaliplatin	27 (100%)
Irinotecan	27 (100%)
Irradiated organs in the BRICS regimen, n (%)	
Pelvic cavity	1 (3.7)
Lymph nodes	3 (11.1)
Retroperitoneum	1 (3.7)
Liver	14 (51.9)
Brain	2 (7.4)
Spleen	1 (3.7)
Bone	1 (3.7)
Lung	4 (14.8)
Tyrosine kinase inhibitor in the BRICS regimen, n (%)	
Anlotinib	6 (22.3)
Apatinib	2 (7.4)
Regorafenib	11 (40.7)
Fruquintinib	7 (25.9)
Lenvatinib	1 (3.7)
Immunotherapy agents in the BRICS regimen, n (%)	
Nivolumab	3 (11.1)
Sintilimab	7 (25.9)
Toripalimab	7 (25.9)
Serplulimab	1 (3.7)
Cadonilimab	2 (7.4)
Protilimab	2 (7.4)
Camrelizumab	5 (18.6)
Number of BRICS regimen treatment cycles, n (%)	
6	10 (37.0)
<6	17 (63.0)

ECOG PS, eastern cooperative oncology group performance status. PD-1, programmed cell death protein 1. BRICS, Bifidobacterium supplementation, Radiotherapy, Immunotherapy (PD-1 inhibitors), Chemotherapy, and Stereotactic approach.

### 3.2 Clinical outcomes

**Figure 2:**
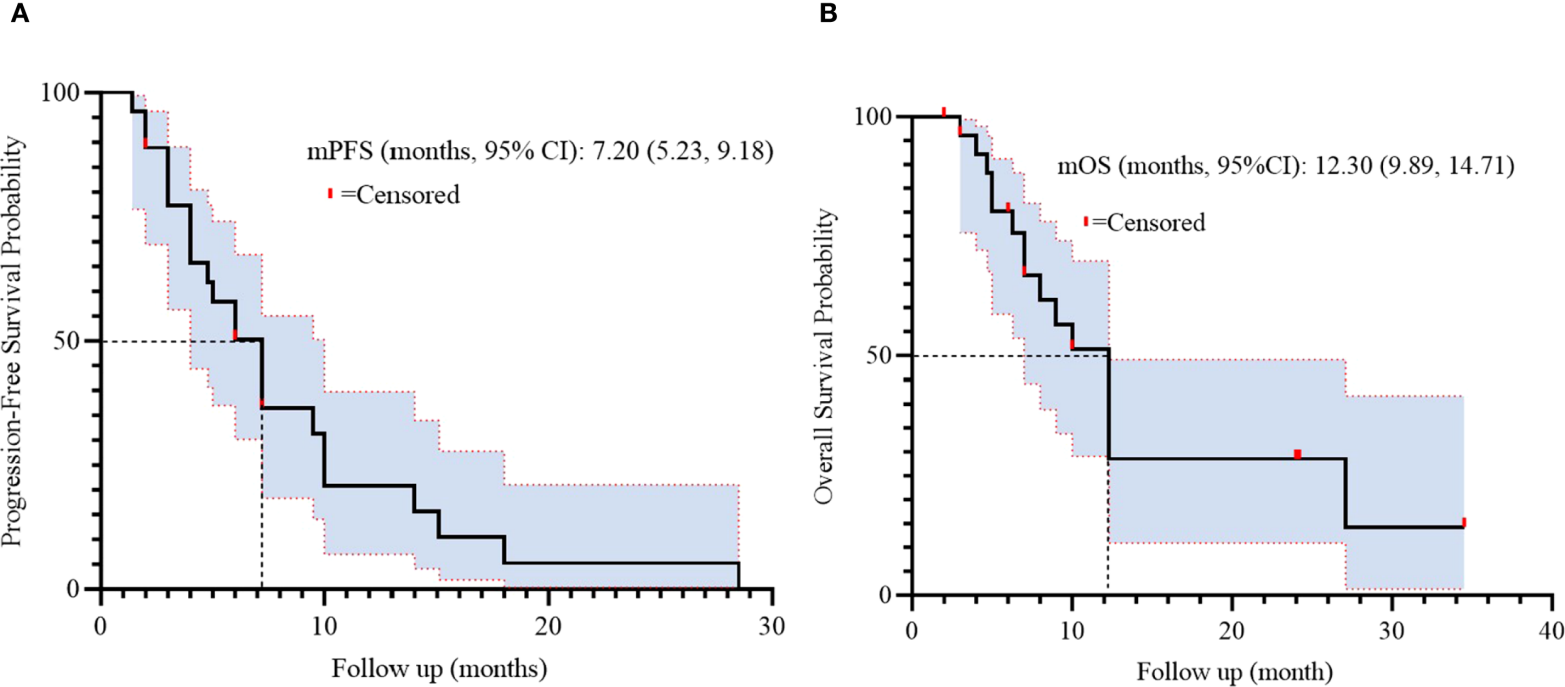
Kaplan-Meier survival curves of PFS **(A)** and OS **(B)** in 27 patients.

Treatment efficacy outcomes are detailed in **Table 2**. Among 27 evaluable patients, partial response (PR) was achieved in 9 patients (33.3%), while stable disease (SD) was observed in 15 patients (55.6%). Progressive disease (PD) occurred in 3 patients (11.1%). No patients exhibited complete response (0%). The objective response rate (ORR) was 33.3% (95% CI: 16.5–54.0), and the disease control rate (DCR) reached 88.9% (95% CI: 70.8–97.6). Survival analysis demonstrated a median progression-free survival (PFS) of 7.20 months (95% CI: 5.23-9.18, **Figure 2**) and a median overall survival (OS) of 12.30 months (95% CI: 9.89-14.71, **Figure 2**).

**Table 2:** Efficacy of BRICS regimen in advanced colorectal cancer patients (n = 27).

Efficacy	All patients (n = 27)
Complete response (%)	0
Partial response (%)	9 (33.3)
Stable disease (%)	15 (55.6)
Progressive disease (%)	3 (11.1)
Objective response rate (%, CR, PR)	9 (33.3, 95% CI: 16.5–54.0)
Disease control rate (%, CR, PR, SD)	24 (88.9, 95% CI: 70.8–97.6)
Median progression-free survival (months, 95% CI)	7.20 (5.23, 9.18)
Median Overall Survival (months, 95% CI)	12.30 (9.89, 14.71)

### 3.3 Prognostic factors for PFS and OS

**Figure 3:**
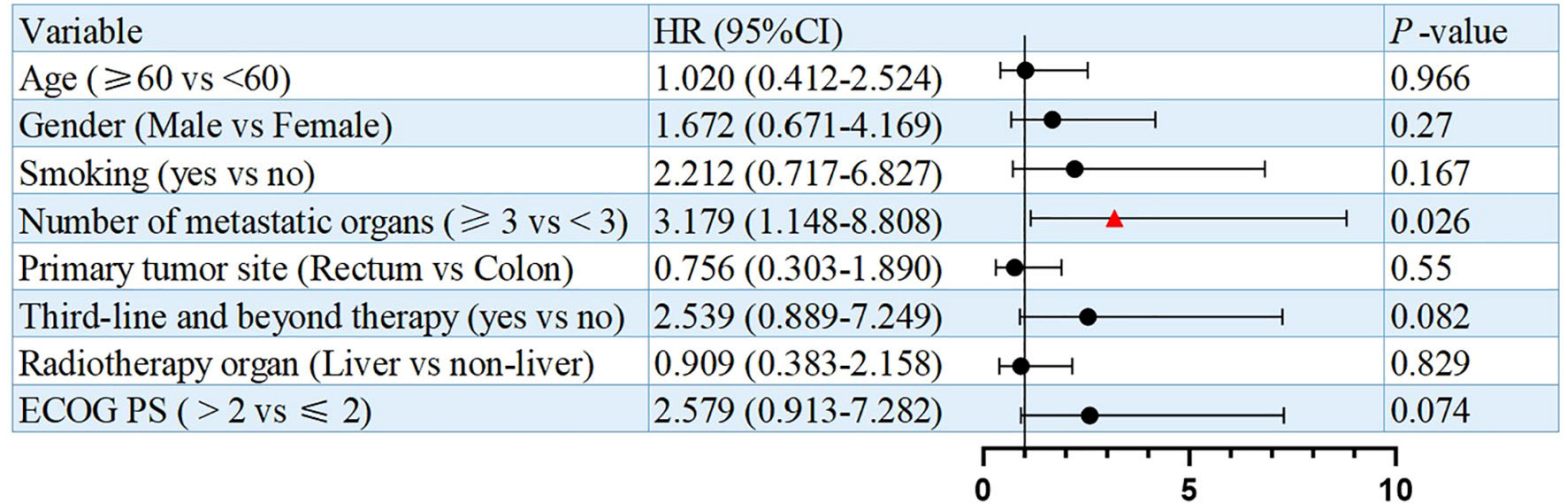
Univariate analysis of prognostic factors for PFS.

**Figure 4:**
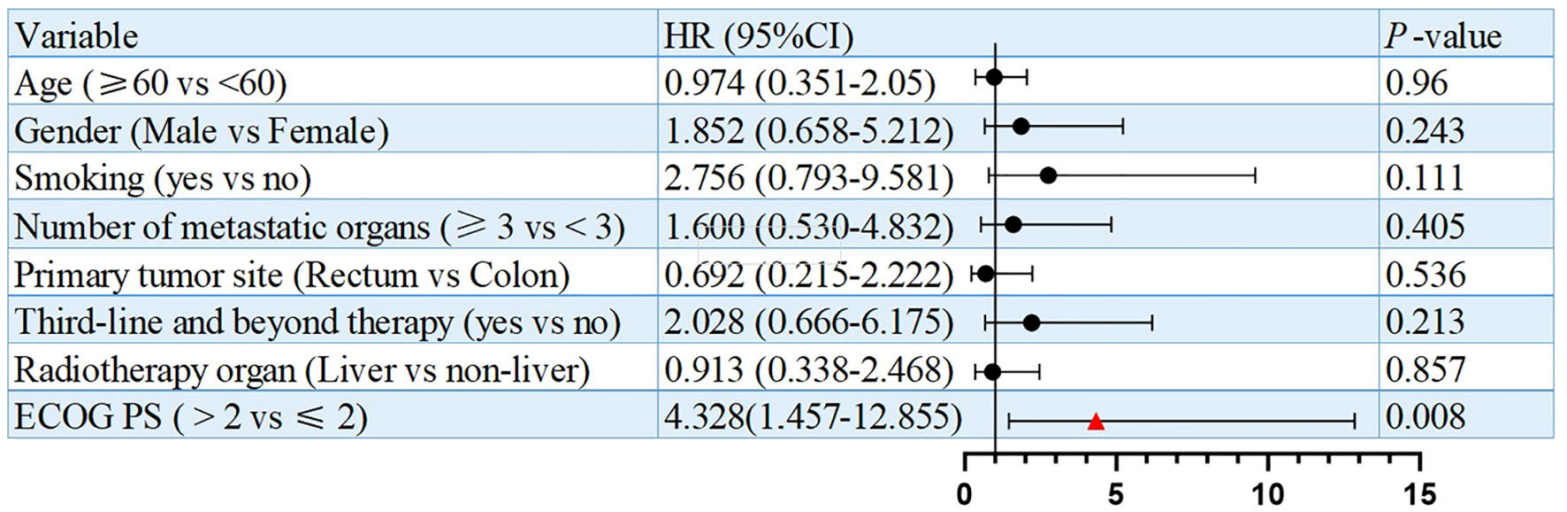
Univariate analysis of prognostic factors for OS.

Univariate analysis identified numbers of metastatic organs (≥3 *vs <*3) as an independent prognostic factor for PFS (HR = 3.179, 95% CI: 1.148-8.808, p=0.026, **Figure 3**). In addition, ECOG PS (ECOG PS ≥2 *vs <*2) was observed as another independent prognostic factor for overall survival (OS) (HR = 4.328, 95% CI: 1.457-12.855, p=0.008, **Figure 4**). Besides, no other variables significantly predicted PFS or OS (all p>0.05, **Figures 3**, **4**).

### 3.4 Subgroup analysis

**Figure 5:**
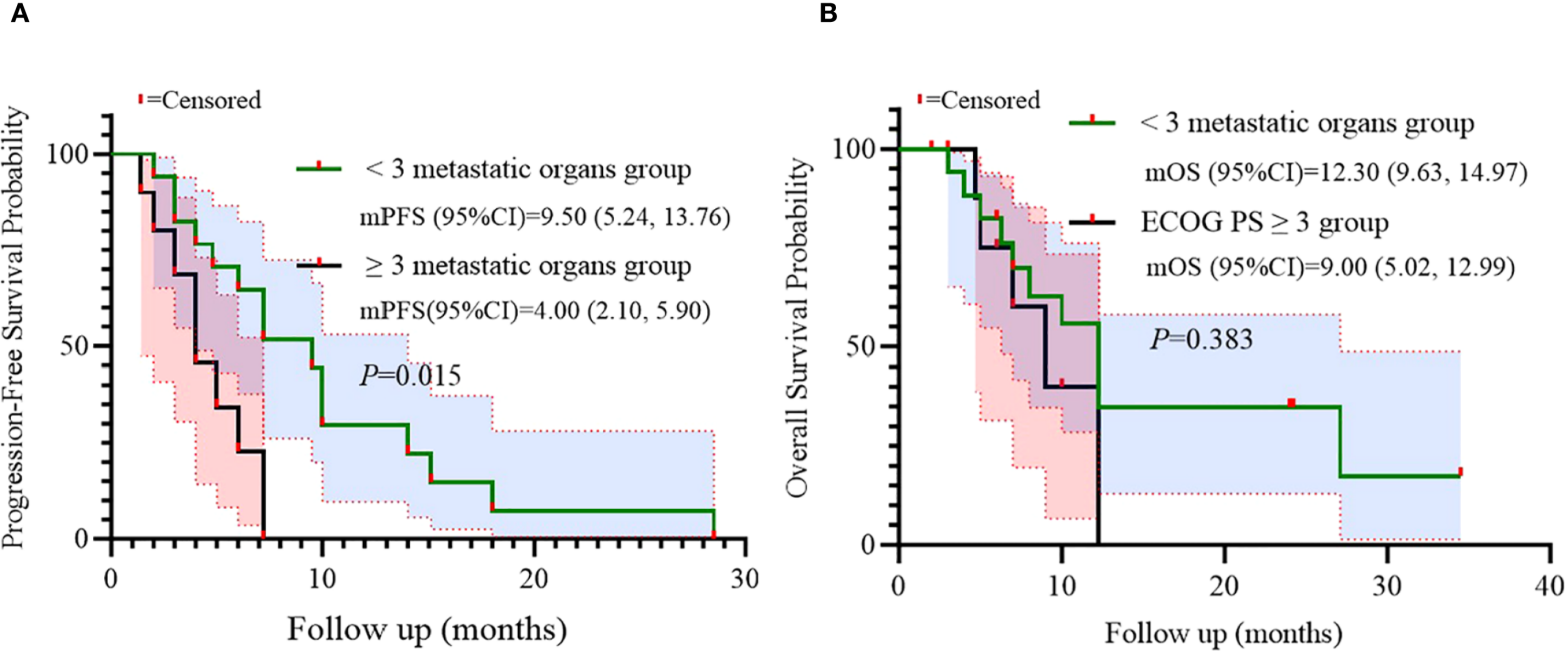
Subgroup analysis of metastatic organs as prognostic factor for PFS **(A)** and OS **(B)**.

Univariate analysis indicated that the number of metastatic organs might be an independent predictor for progression-free survival (PFS). Consequently, survival outcomes were stratified by metastatic burden using a cutoff of 3 organs. A significant difference in PFS was observed between patients with <3 versus ≥3 metastatic organs (log-rank p=0.015), while no statistical difference was found in OS (p=0.383). This suggests that metastatic burden may serve as an independent prognostic factor for PFS in advanced CRC patients receiving salvage BRICS therapy (**Figure 5**).

**Figure 6:**
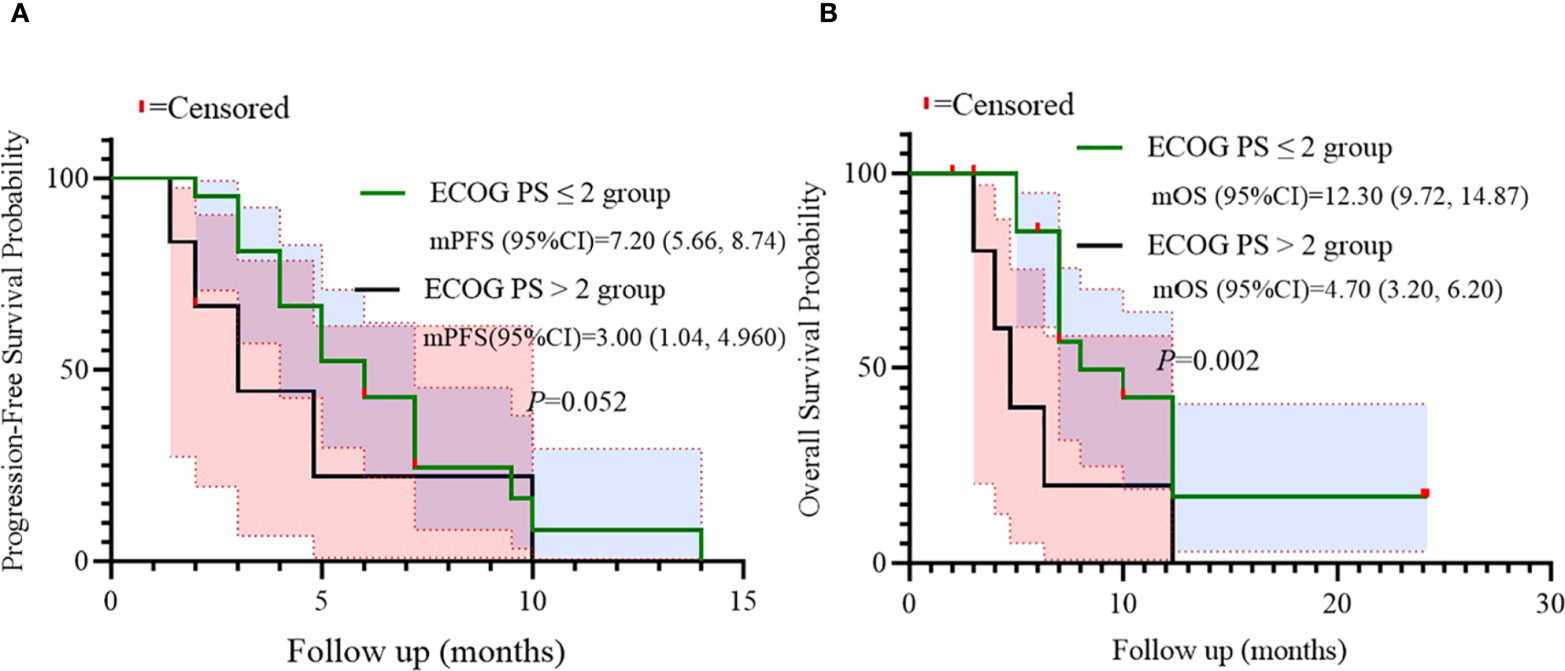
Subgroup analysis of ECOG PS as prognostic factor for PFS **(A)** and OS **(B)**.

Additionally, univariate analysis suggested ECOG PS as a potential independent predictor for OS. Survival outcomes were compared according to ECOG PS (≤2 *vs >*2). A trend toward inferior PFS was observed in patients with ECOG PS >2 (log-rank p=0.052), while a statistically significant difference in OS was found (p=0.002), indicating ECOG PS as an independent prognostic factor for OS in this population (**Figure 6**).

### 3.5 Safety

Treatment-related adverse events are detailed in **Table 3**. The most common grade 1–2 adverse events were fatigue (74.1%), nausea (48.1%), and vomiting (48.1%). Grade 3–4 adverse events included neutropenia (14.8%), thrombocytopenia (3.7%), anemia (3.7%), and diarrhea (3.7%). No grade 5 adverse events or treatment-related deaths occurred.

**Table 3:** Adverse events.

Adverse events		
	Grade 1–2, n (%)	Grade 3–4, n (%)
Diarrhea	5 (18.6)	1 (3.7)
Anemia	11 (40.7)	1 (3.7)
Thrombocytopenia	10 (37.0)	1 (3.7)
Elevated creatinine	9 (33.3)	0
Fatigue	20 (74.1)	0
Nausea	15 (44.4)	0
Vomiting	13 (48.1)	0
Rash	9 (33.3)	0
Neutropenia	14 (51.9)	4 (14.8)
Immune-related pneumonitis	4 (14.8)	0
Immune-related thyroiditis	8 (29.6)	0
Immune-related adrenal insufficiency	6 (22.3)	0
Immune-related myocarditis	5 (18.6)	0
Renal impairment	4 (14.8)	0

## 4 Discussion

This preliminary analysis demonstrates clinically meaningful activity of the BRICS sequential regimen in refractory pMMR/MSS metastatic colorectal cancer (CRC), a population historically resistant to immune checkpoint inhibitors (ICIs) and conventional salvage therapies. With an objective response rate (ORR) of 33.3%, disease control rate (DCR) of 88.9%, median progression-free survival (mPFS) of 7.20 months, and median overall survival (mOS) of 12.30 months, BRICS compares favorably with historical controls. Historically, refractory pMMR/MSS CRC patients exhibit dismal outcomes, with mOS typically below 10 months after progression on oxaliplatin/irinotecan-based regimens and anti-angiogenic agents like regorafenib or fruquintinib (3). The landmark KEYNOTE-016 trial cemented the futility of ICI monotherapy in this population (ORR = 0 for pembrolizumab), highlighting profound intrinsic resistance (5). In addition, in the FRESCO-2, an international, multicenter, randomized, double-blind, phase 3 study, fruquintinib​ demonstrated a statistically significant and clinically meaningful improvement in OS​​ and ​ PFS​​ compared to placebo in patients with refractory mCRC, with a median OS of 7.4 months (95% CI: 6.7–8.2) versus 4.8 months (95% CI: 4.0–5.8) (HR 0.66; 95% CI: 0.55–0.80; p<0.001) and median PFS of 3.7 months versus 1.8 months (HR 0.32; 95% CI: 0.27–0.39; p<0.001) (14). While our PFS and OS exceeds those reported for regorafenib (15), fruquintinib (14) and trifluridine/tipiracil (16). besides, the ORR (33.3%) represents a substantial improvement over historical benchmarks.

The proposed foundation of BRICS lies in its temporal orchestration of three synergistic immunomodulatory strategies: stereotactic body radiotherapy (SBRT)-induced antigen release, probiotic-mediated microbiome priming, and metronomic chemotherapy-driven immunosuppressive cell depletion. SBRT may trigger immunogenic cell death, releasing tumor neoantigens that enhance dendritic cell activation and T-cell priming (6). This aligns with the PEMBRO-RT trial, where SBRT prior to pembrolizumab doubled ORR in PD-L1-negative NSCLC (36% *vs*. 18% for ICI alone) (7). In our cohort, targeting predominantly hepatic metastases (51.9%) may have facilitated systemic immune activation, though the exact contribution requires further investigation given the lack of correlative biomarker data. Crucially, concurrent high-dose Bifidobacterium/Lactobacillus supplementation potentiates this effect by modulating the gut-immune axis. Seminal work by Gopalakrishnan et al. (8) and Matson et al. (16) established that Bifidobacterium enrichment correlates with enhanced CD8+ T-cell infiltration and dendritic cell maturation, thereby overcoming ICI resistance. Our indefinite probiotic administration likely sustained this benefit, differentiating BRICS from transient microbiome interventions. The third pillar-low-dose chemotherapy (e.g., modified FOLFOX)-selectively depletes regulatory T cells (Tregs) and myeloid-derived suppressor cells (MDSCs) while sparing effector T-cells (12), further reshaping the tumor microenvironment. Although this tripartite approach shows promise, its exact immunological mechanisms warrant further investigation.

Notably, BRICS efficacy in CRC appears numerically lower than in our prior NSCLC cohort (ORR: 33.3% *vs*. 95.7%; mOS: 12.30 *vs*. 32.7 months) (13). ​This difference could be attributed to​ inherent biological differences between malignancies. NSCLC tumors often harbor higher tumor mutational burdens (TMB) than CRC, potentially yielding more immunogenic neoantigens post-SBRT (4). Additionally, CRC’s immunosuppressive microenvironment—enriched with TGF-β, IL-23, and regulatory macrophages (17)-may require more intensive modulation. The observed mOS of 12.30 months (95% CI: 9.89-14.71) remains clinically relevant for this heavily pretreated population, particularly when considering that 70.4% received ≥ third-line therapy.

Univariate and subgroup analyses identified two critical prognostic factors. ECOG PS >2 independently predicted inferior OS (HR = 4.860; p=0.042), with a significant survival disadvantage confirmed in stratified analysis (log-rank p=0.002). Metastatic burden (≥3 organs) independently predicted shorter PFS (HR = 3.179; p=0.026), with stratified analysis confirming significantly reduced PFS (log-rank p=0.015).​​ These findings align with established prognostic models in advanced CRC, and highlight the importance of patient selection (18). Patients with compromised performance status (ECOG PS >2) or high metastatic burden (≥3 organs) may derive limited benefit from sequential multimodality therapy due to reduced physiologic reserve and aggressive tumor biology. ​The predominance of liver-directed SBRT (51.9%) warrants mechanistic exploration, as hepatic metastases uniquely influence systemic immunity via liver-resident immunosuppressive cells (19), potentially modulating BRICS efficacy-a hypothesis requiring further investigation.

The safety profile remains manageable despite the extended cohort.​​ Grade 3–4 toxicities occurred in 22.2% of patients, primarily hematologic events (neutropenia: 14.8%; thrombocytopenia: 3.7%; anemia: 3.7%). ​This contrasts favorably with regorafenib’s high incidence of hand-foot syndrome (47%) and fatigue (48%) (15), and trifluridine/tipiracil’s grade ≥3 neutropenia (34%) (16). The absence of radiation-induced liver injury despite predominant hepatic targeting validates our SBRT dosing constraints. While immune-related adverse events were predominantly low-grade, the 14.8% incidence of pneumonitis warrants vigilance in larger studies. Probiotic supplementation may contribute to gut barrier stabilization (11), but larger studies are needed to confirm this potential benefit.

Several limitations warrant acknowledgment. ​First, the retrospective design and modest sample size (n=27) limit statistical power for multivariable analyses. Future larger studies will incorporate this to account for confounders like age and prior therapies. Second, heterogeneity in prior therapies, TKI choices (regorafenib: 40.7%; fruquintinib: 25.9%), and PD-1 inhibitors (toripalimab: 25.9%; sintilimab: 25.9%) may confound efficacy assessments. While this reflects real-world clinical practice, future prospective trials will standardize protocols. Mechanistic validation via scRNA-seq is ongoing. Third, the lack of biomarker data (TMB, TCR clonality, PD-L1, or NGS-confirmed MSI status, fecal microbiome) precludes mechanistic validation of immune conversion. Fourth, 63.0% of patients received <6 BRICS cycles, potentially underestimating the regimen’s full therapeutic potential.

In conclusion, this preliminary analysis suggests​ that BRICS ​may offer​ a clinically meaningful approach for refractory pMMR/MSS metastatic CRC. ​However,​​ future prospective randomized trials will standardize protocols to validate these findings, as proposed in our ongoing research plan. Ongoing translational studies, including pre- and post-treatment tumor biopsies and fecal microbiome sequencing, will empirically validate these mechanisms in our prospective cohort. In addition, future prospective randomized trials with correlative biomarker studies are ​essential​ to validate these observations and elucidate the underlying mechanisms of action.

## Data Availability

The original contributions presented in the study are included in the article/supplementary material. Further inquiries can be directed to the corresponding authors.
